# The Limitation of the Combination of Transition State Theory and Thermodynamics for the Reactions of Proteins and Nucleic Acids

**DOI:** 10.3390/biom12010028

**Published:** 2021-12-25

**Authors:** Nobuo Shimamoto

**Affiliations:** National Institute of Genetics, Mishima, Shizuoka-ken 411-8540, Japan; nshima@nig.ac.jp; Tel.: +81-90-2139-1019

**Keywords:** chemical ratchet, kinetics, reaction theory, rate equation, transition-state theory, detailed balance, thermal equilibrium

## Abstract

When a reaction is accompanied by a change with the speed close to or slower than the reaction rate, a circulating reaction flow can exist among the reaction states in the macroscopic stationary state. If the accompanying change were at equilibrium in the timescale of the relevant reaction, the transition-state theory would hold to eliminate the flow.

The biochemical reactions of macromolecules are usually analyzed by rate equations similar to the reaction of small molecules. The foundation of describing a reaction by rate equations is generally provided from statistical mechanics, but in some textbooks, it is alternatively introduced as transition state theory combined with thermodynamics because understanding the statistical foundation requires knowledge of the master equation and its further development [1], which is not an easy concept for students of biology.

The alternative introduction has an assumption that all the transition states are equilibrated with the ground states, which is not involved in the statistical foundation. The following kinetic rules are derived from this limiting assumption: (1) a reaction can be described with a single set of rate equations; (2) a detailed balance of reaction holds at equilibrium; (3) the circulating flow among reaction states is prohibited; and (4) the affinity of binding is independent of the binding pathways. These rules do not necessarily hold on the statistical foundations [1].

However, these rules tend to be misunderstood as general thermodynamic rules irrespective of the need for the thermal equilibria of the transition states, especially in experimental biology. About 40 years ago, the interpretation of the observed coupling between dimerization and a conformational change of yeast enolase was attacked because of the absence of detailed balance, but the debate became fruitless because of the absence of the discussion on the timescales of the coupling reactions [2,3,4]. Furthermore, it tends to be denied that an affinity between a protein and its specific site depends on the DNA length harboring the site by the pathway of one-dimensional diffusion of a protein along the DNA. Thus, experimental evidence suggesting the contribution of one-dimensional diffusion was ignored (Figure 10 of Ref. [5] and reported with a declared reservation of the “violation of the thermodynamic rule” [6]. Recently, Lukatsky and his colleagues made a series of genomic analyses of the binding sites for protein factors and found that the functional sites in vivo are determined not only by their specific DNA sequences but also by homo-multimeric and repetitive sequences located in the vicinity but with a distance from the complex [7,8,9,10]. The finding suggests the possible contribution of one-dimensional diffusion because such sequences are known to promote one-dimensional diffusion [11]. However, they were reluctant to clearly describe the mechanism, probably being concerned about likely rejections.

Similarly, the publications of experimental evidence for the contribution of one-dimensional diffusion [12] took this author decades because of the superstition about the generality of detailed balance. The validity of the transition-state foundation depends on whether the molecular movements in a transition state are much faster than the relevant reaction. When there is a conformational change with the timescale of the same order as or slower (longer) than that of the relevant reaction, and when the conformations show different reactivities of the relevant reaction, the reaction coordinate and the slow conformational change must be simultaneously considered in the kinetic analysis. Otherwise, a reactant becomes inhomogeneous in terms of its reactivity. In that case, the transition-state foundation collapses in the timescale because of the lack of equilibrium of the conformational change [1]. If the conformational forms of different reactivities were grouped into a reactant with population-averaged reactivity, its value should keep changing according to the progress of the conformational change, making it impossible to define a time-independent rate constant in the timescale of the relevant reaction. In such a case, a reactant must be considered as an ensemble of reactant molecules that switch their reactivities according to their conformational forms. This reaction mechanism converges into a stationary state because the molecular switchings occur at random phases. The stationary state thus includes microscopic non-equilibrium as the switchings, and the absence of a microscopic equilibrium makes the rule of detailed balance indifferent. This is the case observed for the binding between *E. coli* TrpR and *trpO*, and the mechanism was named chemical ratchet [12,13,14].

This mechanism is described as the switching sets of rate equations and works as a ratchet to generate a circulating flow prohibited by detailed balance based on the transition-state foundation. The flow is allowed under the statistical foundation, and its direction is determined by the rate constants. The relationship among the constants making the flow zero is called the Kolmogorov criterion. It is equivalent to the detailed balance (Supplementary of Ref. [12]) and non-obligatory in the statistical foundation. In other words, a detailed balance of reaction looks absolute if the transition-state foundation is assumed, but the balance does not hold in the case of a chemical ratchet.
